# Experiment and Numerical Simulation for the Compressive Buckling Behavior of Double-Sided Laser-Welded Al–Li Alloy Aircraft Fuselage Panel

**DOI:** 10.3390/ma13163599

**Published:** 2020-08-14

**Authors:** Yunlong Zhang, Wang Tao, Yanbin Chen, Zhenkun Lei, Ruixiang Bai, Zhenglong Lei

**Affiliations:** 1 State Key Laboratory of Advanced Welding and Joining, Harbin Institute of Technology, Harbin 150001, China; taowang81@sina.com (W.T.); leizk@dlut.edu.cn (Z.L.); bairx@dlut.edu.cn (R.B.); leizhenglong@hit.edu.cn (Z.L.); 2 State Key Laboratory of Structural Analysis for Industrial Equipment, Dalian University of Technology, Dalian 116024, China

**Keywords:** compressive buckling, double-sided laser-welded panel structure, fringe projection profilometry, phase-unwrapping with multi-frequency fringes, finite element method, aluminum–lithium alloy

## Abstract

The aim of this work was to study the buckling behavior and failure mode of the double-sided laser-welded Al–Li alloy panel structure under the effect of axial compression via experimental and numerical simulation methods. In the test, multi-frequency fringe projection profilometry was used to monitor the out-of-plane displacement of the laser-welded panel structure during the axial compression load. In addition, the in-plane deformation was precisely monitored via strain gauge and strain rosette. The basic principles of fringe projection profilometry were introduced, and how to use fringe projection profilometry to obtain out-of-plane displacement was also presented. Numerical simulations were performed using the finite element method (FEM) to predict the failure load and buckling modes of the laser-welded panel structure under axial compression, and the obtained results were compared with those of the experiment. It was found that the fringe projection profilometry method for monitoring the buckling deformation of the laser-welded structure was verified to be effective in terms of a measurement accuracy of sub-millimeter level. The structural failure was caused by local buckling of the skin. The observed failure modes such as local buckling of the skin, bending deformation of the stringers, continuous fracture of several welds, and failure of local strength and stiffness were attributed to the deformed laser-welded panel structure under the axial compression. The predicted failure load in the numerical simulation was slightly smaller than that of the experimental test, and the error of the simulation result relative to the test result was −2.7%. The difference between them might be due to the fact that the boundary and loading conditions used in the FEM model could not be completely consistent with those used in the actual experiment.

## 1. Introduction

Laser beam welding (LBW) was proven to be an effective technique for manufacturing of the aircraft fuselage panels fabricated by heat-treatable aluminum alloys on account of its low distortion and excellent mechanical properties [1]. The joints made using LBW can reduce the weight of the aircraft fuselage panels; consequently, the final transportation costs are decreased due to a lack of rivets and sealant compared with the traditional riveting technology [2,3,4]. Moreover, welding takes less manufacturing time than mechanical fastener assembly, thus reducing the manufacturing costs [5,6]. As an alternative to the conventional aluminum alloy, the aluminum–lithium (Al–Li) alloy with low density, high elasticity modulus, high specific stiffness, and high specific strength can be used in welding as the panel materials for the aircraft fuselage [7,8].

The LBW technique is more suitable for optimizing the complex geometry of structures of the aircraft fuselage panel in terms of mechanical stiffness, strength, production speed, and visual quality [4] compared to low-energy heat input solid-state joining processes like friction stir welding (FSW). The LBW technology was already applied in a limited number of larger metallic aircrafts by Airbus [5,9] with limited industrialization experience and limited knowledge on in-service performance. In recent years, several studies were performed on the weld formations, microstructure, mechanical properties, porosity, and cracking susceptibility of laser-welded joints of aluminum alloy with or without a single stringer in order to understand the degradation of the local joint materials and defects due to welding [10,11,12,13,14,15]. However, little direct research in the public domain examined the buckling and post-buckling behavior of laser-welded Al–Li alloy panel structures with several welded stringers under compressive load [16,17,18].

Because of the complicated stress and deformation in the actual engineering structure, it is important to explore the compressive buckling behavior of the laser-welded panel structure. The laser-welded panel structure consists of a thin skin and several stringers with a simple cross-section. The skin and stringer are effectively connected by the laser welding process, and the connection part is called the weld. The failure modes of the laser-welded structure with a single stringer under axial compression include deformation of the skin (as well as the stringer), weld fracture, and strength reduction [19]. For a laser-welded structure with several stringers, axial compression is more likely to cause local wrinkling and buckling of skin, longitudinal bending deformation or even collapse of stringers, continuous fracture of several welds, and local strength and stiffness failure. Therefore, the buckling modes and the evolution of panel structures with several stringers under axial compression are more complicated [16,17,18].

The evolution of buckling mode is the basic feature of the deformation of the panel structure. So far, many studies on the buckling and post-buckling behavior of aluminum alloy panel structures under compression [16,17,18], shear [20,21], and combined compression–shear loads [22] were carried out. In order to improve the bearing capacity of aluminum alloy-based panel structures, it is necessary to establish numerical models to predict the buckling performance of the panel structures and to optimize the design of panel structures. It is also vital to have adequate experimental data in order to verify these models. The experimental data mainly refer to in-plane deformation and out-of-plane displacement during buckling and post-buckling processes. The former can be monitored by strain gauges [21,22,23], while the latter is mainly monitored by full-field and noncontact optical techniques, such as Moiré interferometry patterns [24], digital speckle technology [25], and the fringe projection profilometry (FPP) method [23]. The quantitative data of plane deformation and buckling modes can be easily obtained and displayed using full-field and noncontact optical techniques. By comparing the experimental results of optical measurement with the results of numerical model calculation, the numerical model can be corrected for further accurate prediction.

The buckling and post-buckling behaviors of the laser-welded panel structures under axial compression were verified by employing experimental and numerical analysis. In this study, the basic principles of FPP, the phase-shifting technique, and the phase-unwrapping technique with multi-frequency fringes were comprehensively described in the numerical analysis. In order to record the in-plane strain state and the out-of-plane evolution of the buckling, a strain gauge and the FPP method were utilized, respectively. The numerical model to analyze the buckling and post-buckling processes of the laser-welded panel structure was established. Specifically, the buckling displacements were measured by the full-field and noncontact optical technique and compared with those calculated by numerical simulation.

## 2. Basic Principles

### 2.1. Fringe Projection Profilometry Method

**Figure 1:**
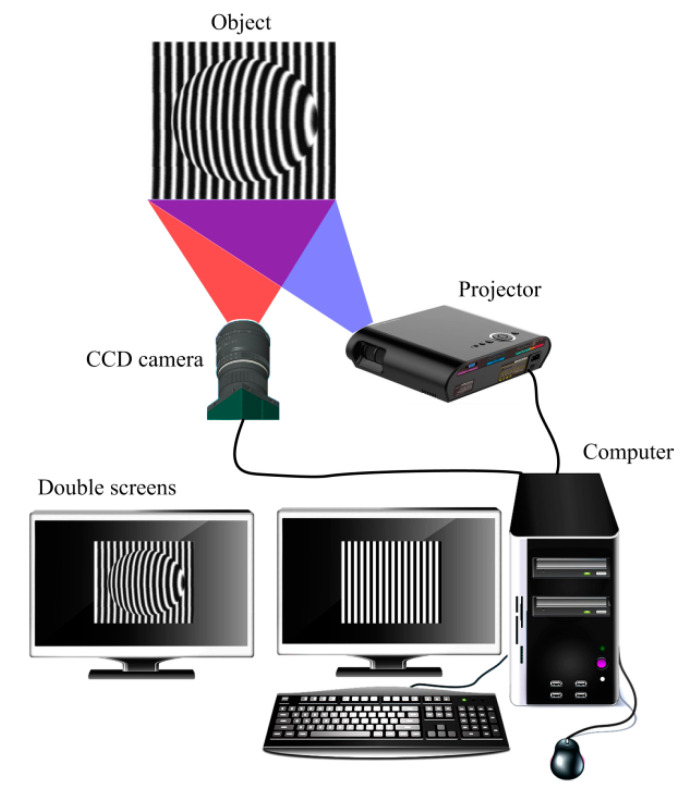
Fringe projection profilometry using double computer screens.

The sinusoidal periodic fringes were simulated by computer and projected onto the surface of the object with the help of a projector. Thereafter, the information related to the height of the object was modulated into the distorted fringes. The distorted fringes recorded by a charge-coupled device (CCD) camera were displayed on the computer screen. Finally, the distorted fringes were further demodulated and reconstructed using height information. Figure 1 shows the schematic diagram of the fringe projection profilometry. The distorted fringe on the surface of the object was obtained by the CCD camera and displayed on the computer screen, as can be seen in Figure 1.

The sinusoidal periodic fringes are projected on the surface of the measured object, and the intensity of the distorted fringes can be expressed as Equation (1):

(1) I(x,y)=A(x,y)+B(x,y)cos[2πfx+ϕ(x,y)],

where *A*(*x*, *y*) and *B*(*x*, *y*) are the background light component in the *XY*-direction and surface reflectivity, respectively. The variable *f* is the spatial frequency of the fringes projected on the reference plane. The phase *φ*(*x*, *y*) represents the phase of the object at a height of *H*(*x*, *y*). When the object is moved away from its initial position, the sinusoidal periodic fringes projected on the reference plane can be expressed as Equation (2):

(2) $$I_{0}(x,y)=A(x,y)+B(x,y)\cos[2\pi fx+\phi_{0}(x,y)],$$

where the phase *φ*_0_(*x*, *y*) is the initial phase of the reference plane. Thus, the phase difference between the object plane and the reference plane can be expressed by the following Equation (3):

(3) $$\Delta \phi (x,y)=\phi (x,y)-\phi_{0}(x,y).$$

Generally, the relationship [26] between the height *H*(*x*, *y*) and phase difference Δ*φ*(*x*,*y*) can be expressed as follows Equation (4):

(4) $$H(x,y)=\frac{L\cdot \Delta \phi (x,y)}{2\pi fD+\Delta \phi (x,y)},$$

where *L* and *D* are the distance from the CCD camera to the reference plane and the distance from the CCD camera to the projector, respectively. According to polynomial expansion, the relationship between the height and phase difference can be rewritten as Equation (5):

(5) $$H(x,y)=\sum_{i=0}^{\infty }C_{i}\cdot \Delta \phi^{i}(x,y),$$

where unknown constant *C_i_* (*i* = 0, 1, 2, ...) can be obtained using a series of experimental height data.

### 2.2. Phase-Shifting Method

By applying an *N*-step phase-shifting method (PSM), the intensity of the *i*-th phase-shifted fringe can be calculated from Equation (1) as Equation (6):

(6) $$I_{i}(x,y)=A(x,y)+B(x,y)\cos[2\pi fx+\phi (x,y)+(i-1)\cdot \frac{2\pi }{N}],$$

where *N* is an integer, *N* ≥ 3, and *i* = 1, 2, ..., *N*.

**Figure 2:**
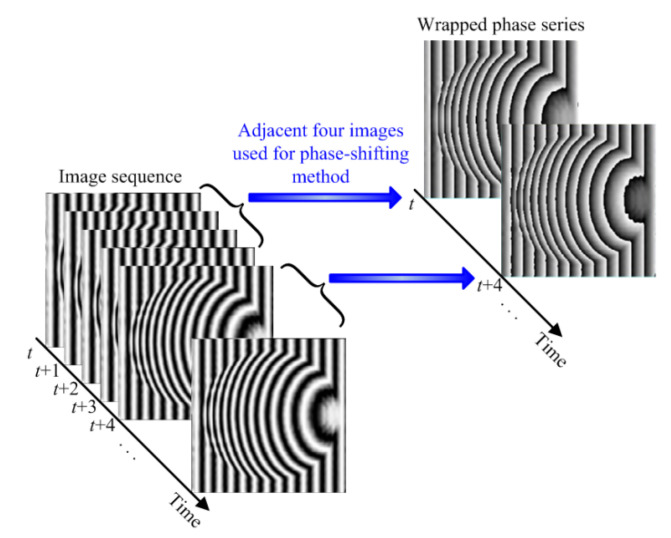
Procedure of the four-step phase-shifting method showing the four adjacent images, a sequence of phase-shifted images captured by the charge-coupled device (CCD) camera, and the corresponding wrapped phase.

For a four-step PSM (*N* = 4), four sinusoidal fringes with initial phases $(i-1)\cdot \frac{2\pi }{N}$ are projected continuously on the surface of the object with an equal time interval. The distorted fringes are simultaneously captured by the CCD camera. The speed rate of the projection is defined similar to the capturing rate of the CCD camera. As shown in Figure 2, four adjacent images in the sequence of multiple images are used for the four-step PSM. The four phase-shifted fringes can be rewritten from Equation (6) as follows Equation (7):

(7) $$\{\begin{array}{l} I_{1}(x,y)=A(x,y)+B(x,y)\cos[2\pi fx+\phi (x,y)]\\ I_{2}(x,y)=A(x,y)+B(x,y)\cos[2\pi fx+\phi (x,y)+\pi /2]\\ I_{3}(x,y)=A(x,y)+B(x,y)\cos[2\pi fx+\phi (x,y)+\pi ]\\ I_{4}(x,y)=A(x,y)+B(x,y)\cos[2\pi fx+\phi (x,y)+3\pi /2] \end{array}.$$

Consequently, the wrapped phase can be obtained from the below-mentioned Equation (8).

(8) $$\phi^{w}(x,y)=\arctan[\frac{I_{4}(x,y)-I_{2}(x,y)}{I_{1}(x,y)-I_{3}(x,y)}],$$

where the wrapped phase *φ*^w^(*x*, *y*) has a value in the range of [−π, π) using the arctan[] function.

### 2.3. Phase-Unwrapping Method with Multi-Frequency Fringes

Due to the variation in the thickness of the object composed by a complex shape, the phase of the projected fringe measured out between the different positions at the surface of the object is usually discontinuous. In order to determine the phase correctly, the discontinuity in the phase of the fringe must be precisely detected. In general, FPP technology uses the phase-unwrapping method with multi-frequency fringes (PUM) to obtain the full-field phase distribution of the projected fringe. In order to get the real phase distribution, it is necessary to unwrap the wrapped phase obtained from the 3D object. In practical applications, multi-frequency fringes can be used to realize fast and precise unwrapping of the phase in the case of discontinuous fringes [27].

By using fringes of various frequencies for PUM, the full-field unwrapped phase distribution can be determined automatically and quickly. The fringe with low frequency can produce the full-field phase distribution. However, the unwrapping phase distribution of the high-frequency fringe can be easily calculated without unwrapping of the general phase. The unwrapped phase with the fringe frequency *f_h_* is obtained [28] as Equation (9).

(9) $$\phi_{h}{}^{u}(x,y)=\phi_{h}^{w}(x,y)+2\pi \cdot \mathrm{Round}[\frac{\left(f_{h}/f_{l})\cdot \phi_{l}^{u}(x,y)-\phi_{h}^{w}(x,y\right)}{2\pi }],$$

where the subscripts *h* and *l* represent the projected fringe of *h*-th and *l*-th with high frequency of *f_h_* and low frequency of *f_l_*, respectively. The superscripts u and w indicate the unwrapped and wrapped phase, respectively. The Round[] function is defined to obtain the closest integer value. It can be noted that the unwrapped phase of *φ_l_*^u^(*x*, *y*) is equal to the wrapped phase of *φ_l_*^w^(*x*, *y*) when the low frequency of *f_l_* and the Round[] function are 1 and 0, respectively.

**Figure 3:**
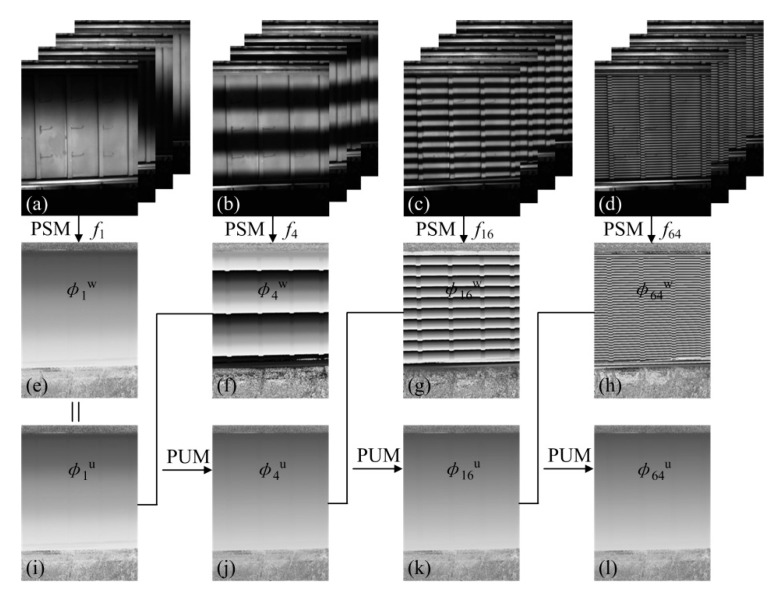
Flowchart for the phase-unwrapping method with multi-frequency fringes. (**a**–**d**) Four groups of distorted fringes with different frequencies of 1, 4, 16, and 64. (**e**–**h**) The corresponding wrapped phase *φ_h_*^w^(*x*, *y*) was obtained by using the four-step phase-shifting method (PSM). (**i**) The unwrapped phase *φ*_1_^u^(*x*, *y*) is similar to its wrapped phase *φ*_1_^w^(*x*, *y*). (**j**) The unwrapped phase *φ*_4_^u^(*x*, *y*) was obtained by combining (**f**) and (**i**) via the phase-unwrapping method (PUM). (**k**,**l**) The unwrapped phases *φ*_16_^u^(*x*, *y*) and *φ*_64_^u^(*x*, *y*) were obtained from a step similar to (**j**).

The PUM is explained by a four-step PSM at the different fringe frequencies, as shown in Figure 3. Four groups of four-step phase-shifting fringes with frequencies *f_h_* of 1, 4, 16, and 64 are generated by the computer and projected in turn with equal time intervals onto the laser-welded panel structure consisting of a discontinuous surface. The corresponding distorted fringes are captured synchronously, as seen in Figure 3a–d. The four calculated wrapped phases *φ*_1_^w^(*x*, *y*), *φ*_4_^w^(*x*, *y*), *φ*_16_^w^(*x*, *y*), and *φ*_64_^w^(*x*, *y*) with different frequencies of 1, 4, 16, and 64 utilizing a four-step PSM (Equation (8)) are displayed in Figure 3e–h. As can be seen in Figure 3e,i, for the first projected fringe with the frequency *f*_1_ of 1, the unwrapped phase is similar to the wrapped phase, i.e., *φ*_1_^u^(*x*, *y*) = *φ*_1_^w^(*x*, *y*). For the frequency *f*_4_ of 4, the unwrapped phase *φ*_4_^u^(*x*, *y*) in Figure 3j can be calculated according to the PUM (Equation (9)) by combining the wrapped phase *φ*_4_^w^(*x*, *y*) and the unwrapped phase *φ*_1_^u^(*x*, *y*). Furthermore, the PUM flowchart of the unwrapped phases *φ*_16_^u^(*x*, *y*) and *φ*_64_^u^(*x*, *y*) of the related frequency *f*_16_ and *f*_64_ of 16 and 64 is similar to that of the unwrapped phase *φ*_4_^u^(*x*, *y*). In the actual measurement, only the unwrapped phase *φ*_64_^u^(*x*, *y*) for the highest fringe frequency *f*_64_ of 64 with the highest accuracy is needed.

## 3. Experiment

### 3.1. Test Specimen

**Figure 4:**
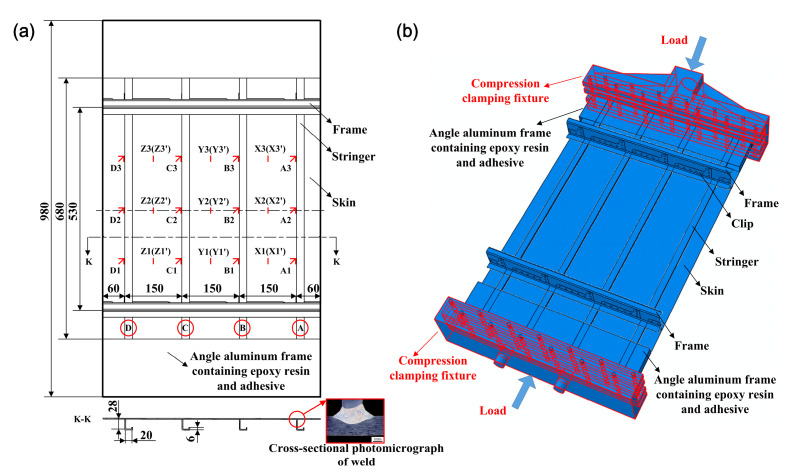
(**a**) Geometry of the double-sided laser-welded panel stiffened by L-section stringers and (**b**) schematic of axial compressive load. The letters A–D represent the four stringers. The labels X1/X1′–X3/X3′, Y1/Y1′–Y3/Y3′, and Z1/Z1′–Z3/Z3′ represent nine pairs of back-to-back strain gauges pasted on the front and back of the panel. The labels A1–A3, B1–B3, C1–C3 and D1-D3 represent 12 strain rosettes pasted on the front of the skin close to the stringers.

The fabricated test specimen consisted of a flat skin (AA 2060-T8, 2 mm thick) stiffened with four longitudinal L-section stringers (AA 2099-T83, 2 mm thick), as illustrated in Figure 4a. The four stringers (namely, A, B, C, and D) were equally spaced with a distance of 150 mm and centered along the longitudinal axis of the panel, as shown in Figure 4a. Each stringer was welded to the skin via double-sided laser welding (DSLW) with ER 4047 Al–Si alloy as the filler metal. The welding parameters were used via reference from the literature [13], as listed in Table 1. No post-weld heat treatment was carried out on the double-sided laser-welded panel stiffened by L-section stringers. The panel was finally machined by wire-electrode cutting and measured at 980 × 570 mm^2^.

**Figure 5:**
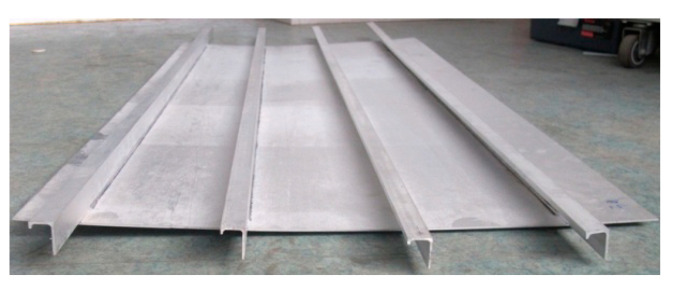
Photograph of the double-sided laser-welded panel which exhibited panel warpage.

The fabricated panel was inspected using non-destructive testing approaches such as visual inspection, dye penetrant inspection, and X-ray detection [29]. All the welds passed the dye penetrant inspection and X-ray detection testing according to the international standard [30]. During visual inspection of the panel, bowing of the stiffeners and warping of the skin along the longitudinal and transverse direction were found, as shown in Figure 5. The laser-welded panel showed significant distortion, which may be due to the simple fixing and clamping device used for laser welding. Similar results were obtained by Hoffman et al. [24] for a friction-stir-welded Al–Cu–Li alloy-based panel structure.

**Figure 6:**
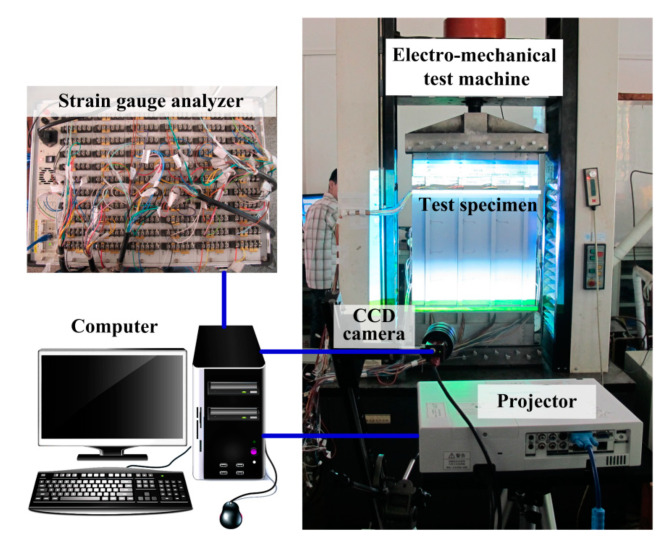
Experimental set-up.

Following the non-destructive inspection, the panel was prepared for further measurement and testing. In order to reduce panel distortion such as column bending generated by load eccentricity induced by longitudinal bowing and transverse warping, appropriate measurements were carried out. It can be seen from Figure 6 that the two frames were centered in the middle of the panel with a spacing of 530 mm. The frames made of 7075-T62 aluminum alloy were connected to several clips via aluminum alloy rivets. The clips made of 7075-T62 aluminum alloy were fastened to the skin and stringers by means of aluminum alloy rivets. The angle aluminum frames were made of 7075-T62 aluminum alloy which contained the potted panel ends with a mixture of 6101 epoxy resin and 600 adhesive. Thereafter, the ends of the potted specimen were machined to be flat and parallel within 0.05 mm.

The resistance strain gauges and strain rosettes were pasted on the surface of the skin to monitor the deformation of the specimen during the loading process. Among them, nine pairs of strain gauges (X1/X1′–X3/X3′, Y1/Y1′–Y3/Y3′, Z1/Z1′–Z3/Z3′) were equally spaced on the front and back of the skin between two frames and between two adjacent stringers, respectively. For instance, two back-to-back strain gauges in the same position of the skin were defined as X1(X1′). The 12 strain rosettes (A1–A3, B1–B3, C1–C3, D1–D3) were equally spaced on the front of the skin close to the stringers between the two frames. Each rosette contained three strain gauges in three different directions (0°, 45° and 90°). In the parallel (0°) and perpendicular (90°) directions of the strain gauges with respect to the stringers in the plane of the panel, rosettes were employed. Taking rosette A1 as an example, 0°, 45°, and 90° strains were defined as A1-0, A1-45, and A1-90, respectively.

The axial compression condition was achieved by using a pair of compression clamping fixtures, as shown by the highlighted red areas in Figure 4b. The specimen was clamped by fixtures at the lower and upper ends in order to limit the longitudinal displacement and axial compressive load, respectively. Both sides of the specimen were restrained by the lateral bolts, which can prevent the out-of-plane displacement of both ends of the panel during compressive loading. In addition, both ends of the panel can be easily managed in the same plane by adjusting the lateral bolts of the fixture, which can also correct the small initial deformation caused by the manufacturing process.

### 3.2. Test Equipment

The experimental test was conducted on an electro-mechanical testing machine (CSS-2250, Changchun tester Co., Changchun, China) with a maximum loading capacity of 500 kN, as shown in Figure 6. During the loading process, the axial load and displacement were measured by the force sensor and the displacement sensor, respectively. The strain data were recorded by the strain gauge analyzer (DH3816, Donghua testing technology Co., Donghua, China).

Four groups of phase-shifted fringes of frequencies 1, 4, 16, and 64 were generated by the computer at an image resolution of 1024 × 768 pixels. The groups of multi-frequency fringes of the test specimen were projected by a projector (TLP-X2000 3LCD, Toshiba, Tokyo, Japan) and collected by a CCD camera (F-080B, Guppy, Stadtroda, Germany) at a speed of 4 fps.

### 3.3. Test Procedure

**Figure 7:**
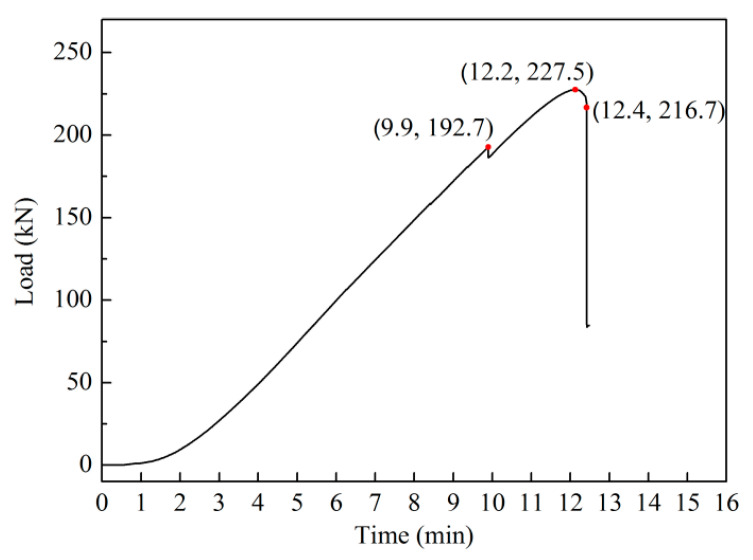
Compressive load history curve.

Under the condition of displacement control, the loading rate was fixed at 0.5 mm/min. The history curve of the compressive load is displayed in Figure 7. The strain gauge analyzer and FPP technology were employed to monitor the strain level and the buckling deflection, respectively. The compressive load vs. time curve is plotted in Figure 7. As can be seen, the compressive load increased gradually with time. After the maximum load reached 227.5 kN, the applied load decreased gradually and finally suffered a sharp collapse at 12.4 min. In addition, the curve changed suddenly at 9.9 min, and a consequent decrease in its value was observed. From the video playback of the loading process and the examination after testing of the panel structure, a rivet connecting the upper frame and a clip was found to have broken suddenly, which reduced the value of the load at 9.9 min.

### 3.4. Strain Measurement

**Figure 8:**
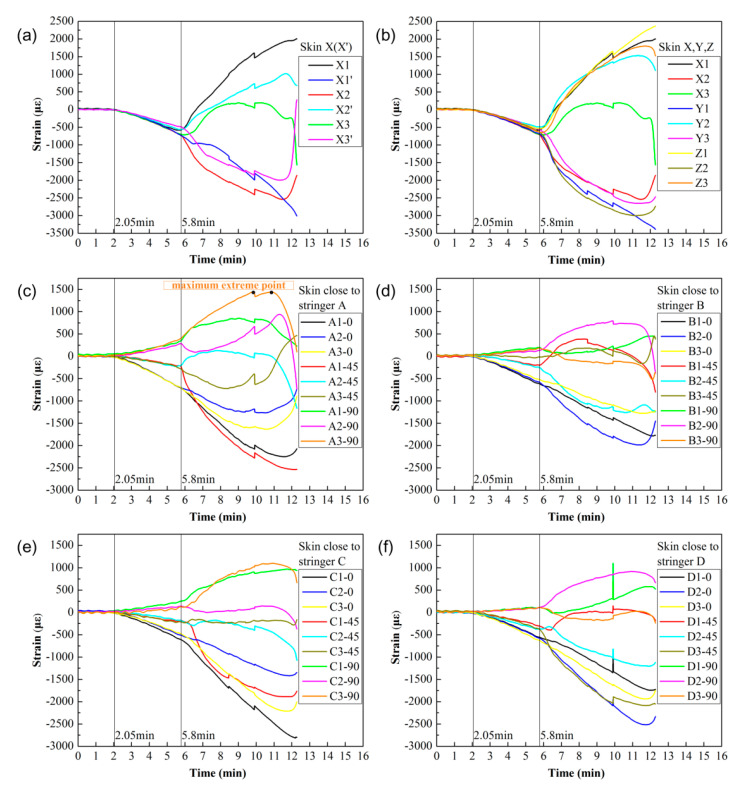
History curves of the strain for the different positions on the skin of the panel: (**a**) front and back of skin X1/X1’–X3/X3’; (**b**) front of skin X1–X3, Y1–Y3, Z1–Z3; (**c**) front of skin A1–A3 close to stringer A; (**d**) front of skin B1–B3 close to stringer B; (**e**) front of skin C1–C3 close to stringer C; (**f**) front of skin D1–D3 close to stringer D.

The history curves of the strain at different positions of the skin on the panel are plotted in Figure 8. The strain gauges and strain rosettes functioned well without any failure during the loading process. The strains of X1 (X1′), X2 (X2′), and X3 (X3′) vs. time and front of the skin (X1–X3, Y1–Y3, Z1–Z3) vs. time curves are plotted in Figure 8a,b, respectively. The values of strain acted like compressive strain (negative value) at the 2.05 min time period. At this time, an obtained compressive load of 10 kN was recorded, as shown in Figure 7. As the compression process progressed, negative values increased. The relationship between load and strain was approximately linear until the time period of 5.8 min. A corresponding compressive load of 100 kN was obtained from the curve (Figure 7). Thereafter, bifurcation of the strain curve occurred on the skin surface. The strain at a few positions such as X1 even became tensile strain (positive value), i.e., the skin no longer had the ability to carry the further compressive load. Therefore, the stringers were supposed to bear the compression load in the subsequent compressive process. Meanwhile, the broken rivet connecting the upper frame and the clip brought about the shake of the strains at 9.9 min.

**Figure 9:**
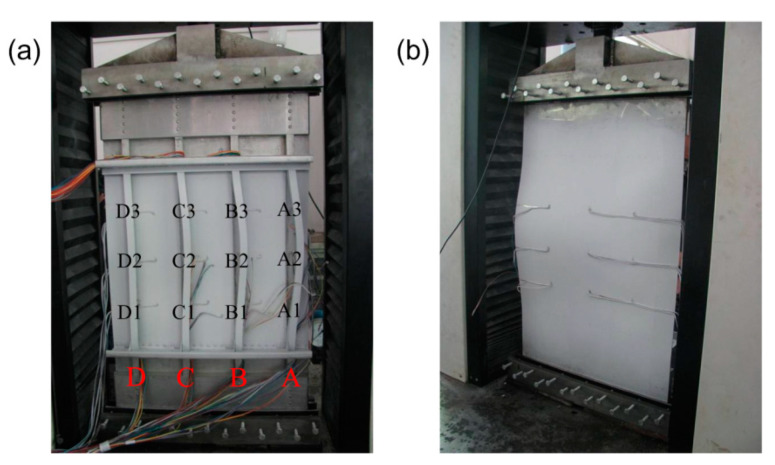
Ultimate collapse images of the panel under the condition that the compressive load was not removed: (**a**) failure image of the front of the panel; (**b**) failure image of the back of the panel.

The strains on the front of the skin close to stringers (A1–A3, B1–B3, C1–C3, D1–D3) are plotted via curves of strain with respect to time in Figure 8c–f, respectively. It can be seen in Figure 8c–f that, at 2.05 min, the strains in the directions of 0° and 45° resulted as compressive strain (negative value), while the absolute value of the slope for the strain in the parallel direction (0°) became larger. However, the strains in the direction of 90° were recorded as tensile strain (positive value), where the welds were subject to tensile stress induced by the compression process in the perpendicular direction (90°). At 5.8 min, the bifurcation of the strain curve occurred on the surface of the skin, which was similar to the strain behavior of X1–X3, Y1–Y3, and Z1–Z3. It is important to note that A3-90 achieved a maximum extreme point of positive value in the direction perpendicular to the stringers in the plane of the panel as compared to the strains at the other positions in the perpendicular direction (90°), as shown in Figure 8c–f. This indicated that the weld close to A3 possessed the maximum tensile stress induced by the compression process as compared to the welds on other positions. This should be considered the main reason for the fracture of the welds and the largest in-plane lateral deformation of stringer A in the perpendicular direction to the weld in the plane of the fractured panel, as compared to that of stringers B, C, and D, as shown in Figure 9.

The appearance of buckling in the panel can be predicted by the bifurcation phenomenon of the strain; however, it was difficult to collect the buckling modes from the strain data. The full-field morphology measured by the FPP technique can be used to distinguish the buckling convexes and concaves.

## 4. Results of Optical Measurement and Analysis

### 4.1. System Calibration

Before the compression test, the fixture was calibrated using strain gauges on the front and back of the panel. When a small amount of compression load was applied to the panel structure, the strain of the corresponding positions on the front and back of the panel was basically the same upon adjusting the position of the panel and filling the gaskets. Later, the applied load was removed.

**Figure 10:**
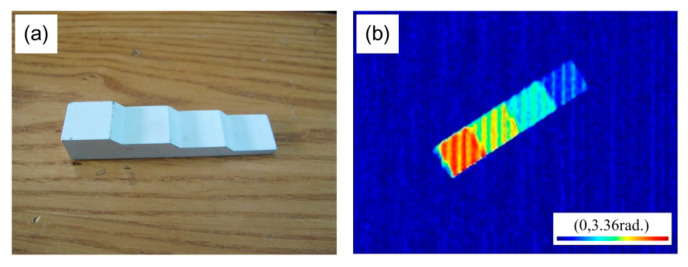
(**a**) The standard test block used in the experiment; (**b**) its full-field phase distribution.

Before setting up the FFP measurement system to test the phase of the specimen, it was necessary to use the standard test block to determine the height–phase relationship and the testing the accuracy of the experimental system. It was crucial to verify the reliability of the projection fringe spacing, projection distance, and imaging system. The standard test block consisted of four steps of 16 mm in total height, and each step was 4.0 mm in height, as shown in Figure 10a. The PSM and PUM methods were used to measure the standard test block. According to the PUM algorithm, the unwrapping phases of the standard test block at the different step positions were obtained, where the reference plane is denoted by 0 mm. The full-field phase distribution of the standard test block is shown in Figure 10b. The phase measurement of the whole standard test block was successful; however, little noise at the connection position of the steps was observed.

**Figure 11:**
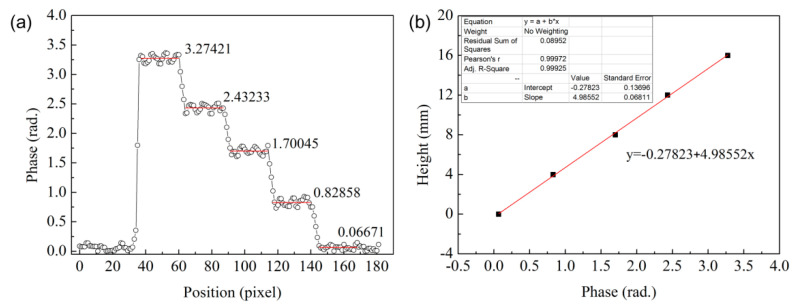
(**a**) The phase of the standard test block; (**b**) the fitting curve showing the height–phase relationship.

The phase of the standard test block was obtained by PUM, as shown in Figure 11a. The five red lines represent the average of the phases with respect to the reference plane and different step positions. The average of the phases of the different step positions were extracted to draw the height–phase relationship, as shown in Figure 11b. The linear relationship between the height and the phase was established, and the correlation coefficient of the linear fitting reached 0.99925. For the data related to the phase of each step position in Figure 11a, the fluctuation range of the phase data did not exceed 0.19 rad. The error in the height was 0.7 mm upon introducing 0.19 rad into the linear equation, as shown in Figure 11b. This indicated that the measurement accuracy of the experimental system reached the sub-millimeter level, which was effective for detecting the buckling deformation of the specimen in this experiment.

### 4.2. Local Buckling Results

**Figure 12:**
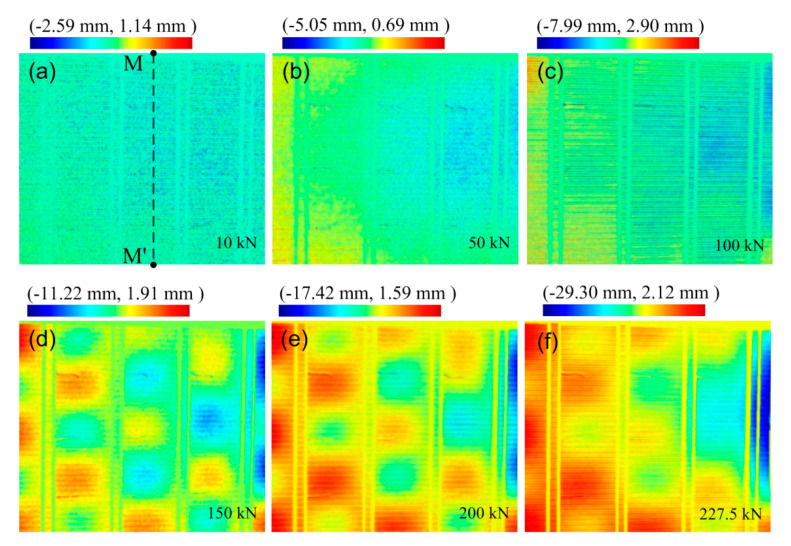
The phase difference evolution of the local buckling with respect to the applied load. The red and blue zones denote convex and concave areas, respectively: (**a**) 10 kN; (**b**) 50 kN; (**c**) 100 kN; (**d**) 150 kN; (**e**) 200 kN; (**f**) 227.5 kN.

During the compressive process, the phase information of the specimen corresponding to different compressive loads before and after deformation were extracted. The phase difference was calculated using Equation (3). By using the relationship of the height and phase, the phase difference was transformed into the deflection information of the specimen under different compressive loads, as shown in Figure 12. It can be found that the evolution of the local buckling was obvious during the process of compression. When the load increased to 10 kN, the skin appeared to have local buckling, which indicated that the skin mainly possessed compressive load. When the load increased to 50 kN, the stringers began to buckle locally, and the deflection of the skin became larger, i.e., the skin and the stringers both possessed compressive load at the same time. Further increment in the load caused further buckling deformation of the skin and stringers. Specifically, a convex or concave distribution due to local buckling of the skin was observed at the loads of 150 kN, 200 kN, and 227.5 kN. Before the maximum of the load, the stringers failed to produce significant in-plane lateral deformation, but out-of-plane deformations of the skin and stringers were discovered in Figure 12. This indicated that the fracture of the welds between the skin and stringers did not occur before the applied maximum load. Compared with the strain measurement, the information of the full-field optical measurement was more comprehensive. Many crucial details that cannot be observed by strain measurement were found in the full-field optical measurement.

### 4.3. Buckling Deflection Curve

**Figure 13:**
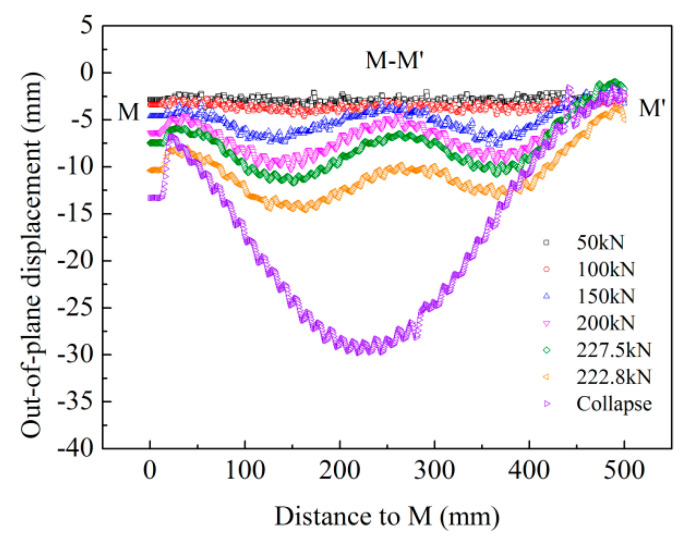
Deflection distributions along the dotted line M–M’ under the different compressive loads in the experiment.

In order to better reflect the changes in buckling deflection of the specimen under different compressive loads, Figure 13 shows the deflection distributions along the dotted line M–M’ in Figure 12 under the different applied loads. It is worth noting that the phase before deformation was subtracted from all these data. Thus, the buckling deflection obtained from the change in phase difference relative to the initial time is shown in Figure 13.

The curve showed wave deformation, indicating that the skin buckled along the dotted line direction. With the increase in compressive load, the value of buckling deflection increased gradually without altering the direction. Comparing the waveforms before and after the collapse of the panel structure, the waveform of the M–M’ line was found to have changed. Three convex and two concave regions were observed along the M–M’ line before the collapse of the panel structure. When the specimen possessed the maximum load of 227.5 kN, the maximum buckling deflection from concave to convex was close to 10.8 mm on the M–M’ line. With the increase in displacement along the load direction, the load on the specimen was decreased, but the buckling deflection continued to increase. The characteristics of local buckling disappeared until the collapse of the panel structure.

### 4.4. Panel Failure Mechanism

Figure 9 shows the ultimate collapse of the panel fabricated by DSLW, following the fracture of the welds between the skin and stringers. By combining this information with the load history curve, strain history curve, and optical measurement data, the failure process can be summarized. Initially, the skin and stringer experienced the initial compressive buckling (Figure 12a and Figure 13) at the time of 2.05 min with a load of 10 kN (Figure 7 and Figure 8). The skin at each marked position shown in Figure 4a possessed compressive strain in the direction of the load (Figure 8). The out-of-plane displacement of the skin and stringer was relatively small (Figure 12) until the load reached 100 kN at 5.8 min. Then, the stringer possessed the post-buckling load after the initial buckling of the skin. Until the applied maximum load of 227.5 kN, the stringers lost their stability to further support the compressive load (Figure 7) because of the bending deformation of stringers (Figure 12). Finally, when the load was unloaded to 216.7 kN (Figure 7), the weld between the skin and stringer A fractured, and the panel structure failed with a continuous fracture of welds between the skin and stringers B, C, and D. The micro-cracks in the position of the weld occurred during the initial buckling, and the micro-cracks gradually increased with the increasing in buckling, which eventually led to the fracture of the weld [19]. The obvious deformation of the skin and stringers is shown in the ultimate collapse images. The in-plane lateral deformation of stringer A in the direction perpendicular to the weld was much larger than that of stringers B, C, and D (Figure 9). Thus, the failure modes of the DSLW panel under axial compression were due to the local buckling of skin, the bending deformation of the stringers, the continuous fracture of several welds, and the failure of local strength and stiffness.

The total deflection data obtained at a different time from the optical measurement were more comprehensive in detail in order to understand the evolution process of local buckling. The basic experimental data of the evolution behavior of the skin in the buckling mode are significant for the analysis of the failure mechanism of the aluminum–lithium alloy panel fabricated by DSLW.

## 5. Buckling Analysis by FEM

### 5.1. FEM Models

**Figure 14:**
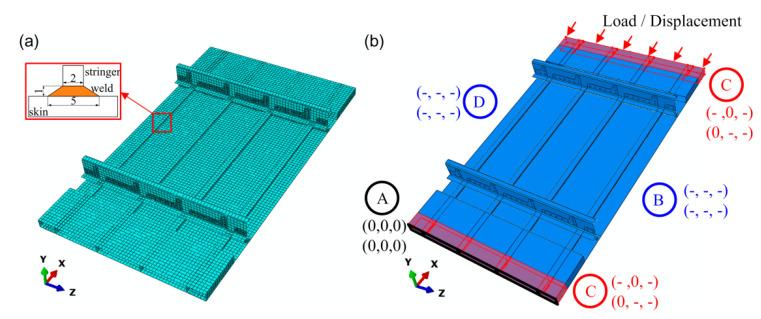
Finite element method (FEM) model for the panel: (**a**) meshing and (**b**) boundary conditions of the model. The two sets of brackets in each region in (**b**) represent the moving degrees of freedom in three axis directions and the rotational degrees of freedom around these three axes. The number 0 means that the direction in (or around) the corresponding axis is restricted while the symbol - means that the direction in (or around) the corresponding axis is free.

The finite element method (FEM) was applied to simulate the compressive test conditions using the ABAQUS finite element analysis software (Version 6.13, Dassault Systèmes Simulia Corporation, Johnston, RI, USA), as shown in Figure 14. The components of the skin, stringer, clip, frame, and angle of the aluminum frame were all modeled using the S4R shell element type. The welds were modeled using the C3D8R solid element type, which was essential to enable both local and global buckling modes of the panel to be simulated [16,31,32]. Tie constraints were employed between the different components. The profile of the weld was idealized as an isosceles trapezoid, as can be seen in Figure 14a. The meshing grid size in the panel structure was 10 mm except for clips, which had a meshing grid size of 4 mm. The boundary conditions were idealized as shown in Figure 14b. Black region A represents the edge of the panel which was limited by fixing all degrees of freedom. Red regions C were clamped by the clamping fixture, for which movement along the *Y*-axis and rotation around the *X-*axis were limited. The movement of region C along the *X*-axis was realized with the edge load of the shell. The blue sides B and D were not constrained, as in the real test.

**Figure 15:**
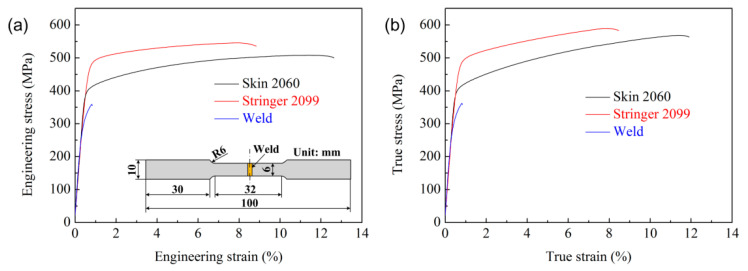
(**a**) Engineering stress–engineering strain curves and (**b**) true stress–true strain curves for the panel materials and the weld.

The material parameters were obtained directly from the tests to improve the model reliability. According to the ASTM E 8M guidelines [33], the tensile strength of the panel materials was tested with a tensile rate of 2 mm/min at room temperature. The tensile strength of the welds achieved from the laser-welded AA 2060 alloy butt joint with ER 4047 filler wire was also tested. Elastic parameters of the panel material are shown in Table 2. The engineering stress versus engineering strain curves of the panel materials and the welds are shown in Figure 15a. The true stress *σ*_true_, true strain *ε*_true_, and plastic strain $\varepsilon_{\mathrm{true}}^{\mathrm{pl}}$ could be expressed based on the engineering stress *σ*_e_ and engineering strain *ε*_e_ (Equations (10)–(12)).

(10) $$\sigma_{\mathrm{true}}=\sigma_{e}(1+\varepsilon_{e}),$$

(11) $$\varepsilon_{\mathrm{true}}=\ln(1+\varepsilon_{e}),$$

(12) $$\varepsilon_{\mathrm{true}}^{\mathrm{pl}}=\varepsilon_{\mathrm{true}}-\varepsilon_{\mathrm{true}}^{\mathrm{el}}=\varepsilon_{\mathrm{true}}-\sigma_{\mathrm{true}}/E.$$

Based on the engineering stress–strain curves, the true material properties were obtained from the engineering values, as shown in Figure 15b. The true stress *σ*_true_ and plastic strain $\varepsilon_{\mathrm{true}}^{\mathrm{pl}}$ could be introduced into software ABAQUS, representing the plastic parameters of the panel materials.

### 5.2. Buckling and Post-Buckling Analysis

In the FEM analysis, the buckling mode of the panel was obtained from the eigenvalue buckling analysis. It could be introduced in the model of the post-buckling analysis, as an initial geometric defect. For the post-buckling analysis, the modified Riks method was used in the present study in order to trace the nonlinear equilibrium path with arc increment length [34]. According to the buckling mode of the actual experiment and the eigenvalue buckling analysis, the sixth-order buckling eigenvalue and the initial deformation size of 2 mm were introduced in the post-buckling analysis. The applied method did not consider the influence of the residual stresses induced by welding and fracture characteristics of the weld on the compressive buckling behavior.

**Figure 16:**
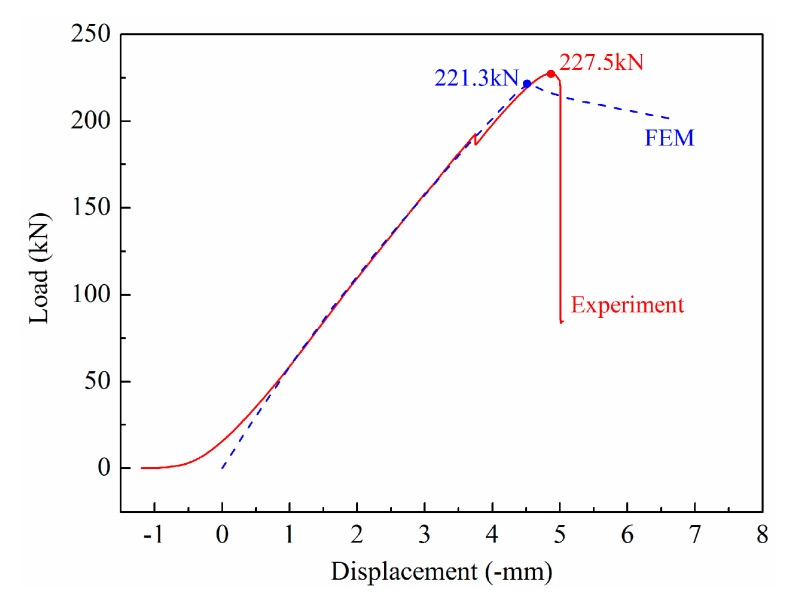
Compressive load and displacement curves for the experiment and FEM model.

The compressive load and displacement curves for the experiment and FEM model are shown in Figure 16. The load and displacement curves for the experiment were translated along the negative displacement axis artificially in order to compare the slopes of these two curves. As a result, the two curves showed a similar slope. This indicated that, in the actual experiment, at the initial stage of the compressive load applied to the panel structure, the load was not evenly distributed along the two ends of the panel structure. With the increase in displacement, the compressive load applied to the panel structure in the experiment was uniform. The variation in the value of the load in the FEM model was basically consistent with the experiment. After the failure loads of the panel, the load in the experiment decreased and suffered a sudden sharp fall, while the load in the FEM model decreased uniformly. This was mainly due to the fact that the factors of weld fracture were not considered in the FEM model. By considering the failure loads for the experiment and FEM model displayed in Table 3, the model predicted the extent of the failure load, which was equivalent to that of a nearly perfect panel structure devoid of residual stresses. The difference between the failure loads of the experiment and FEM model might be due to the reason that the boundary conditions used in the FEM model could not be completely consistent with those used in the actual experiment.

**Figure 17:**
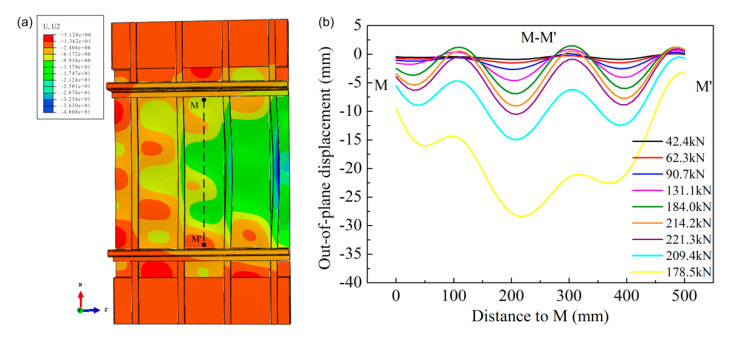
(**a**) The deflection field of the laser-weld panel structure based on the FEM model under failure load; (**b**) the deflection distributions along the dotted line M–M’ under different compressive loads in the FEM model.

Under the failure load, the out-of-plane displacement (deflection) simulated by FEM (Figure 17a) was compared with the result of the experiment in the area between the two frames of the panel structure, as shown in Figure 12f. It can be seen that the same number of convex and concave patterns was obtained by the FEM model and the experiment in the area between each adjacent stringer. The stringer under the failure load condition showed the largest in-plane lateral deformation in the direction perpendicular to the weld in the plane of the panel in the FEM model, which was consistent with that in the test in Figure 9.

To more clearly observe the buckling evolution with load, the deflection distributions along the dotted line M–M’ under different compressive loads were obtained using the FEM model (Figure 17b) and compared with the result obtained by the optical measurement (Figure 13). Because the load data obtained in the modified Riks method were inconsistent with the actual measured data, the deflection distributions could only be analyzed qualitatively. The deflection distribution along M–M’ under different loads in the experiment and the FEM model was approximately the same. The local buckling phenomenon occurred after the applied load of 42.4 kN (50 kN in actual test) and further increased with applied load. The maximum buckling deflection from concave to convex was close to 11.5 mm before the failure of the panel structure; however, a value of 10.8 mm was observed in the actual test. After the collapse of the panel structure in the actual test, the energy was completely released in practice and the buckling was stopped. However, the panel structure was still observed in buckling mode in the FEM model. This is because the factors of weld fracture were not taken into account in the FEM model.

The FEM model can be considered as an idealistic model of analysis; however, the actual laser-welded panel structure showed manufacturing defects and initial damage. In addition, the actual boundary conditions were more complex than those in the FEM model. Therefore, by comparing the FEM results with the test results in terms of failure load, local buckling waveform, wave number, and maximum deflection, it can be concluded that the FEM model and the test results were basically consistent and mutually verified.

## 6. Conclusions

In summary, an experiment and a numerical simulation were used to study the buckling behavior and failure mode of a double-sided laser-welded panel structure under axial compressive load. The finite element model could be utilized to accurately predict the compressive buckling deformation and failure load of the panel structure. The fringe projection profilometry method for monitoring buckling deformation of the laser-welded panel structure was verified to be effective. It showed the advantage of full-field and noncontact measurements. It could be confirmed via a comprehensive analysis of the fractured area that the structural failure was initially caused by local buckling in the skin. Subsequently, the failure modes of the laser-welded panel structure under axial compression were mainly due to local buckling of the skin, bending deformation of the stringers, continuous fracture of several welds, and failure of local strength and stiffness.

## Figures and Tables

**Table 1:** Detailed welding parameters of the double-sided laser welding (DSLW) process.

Welding Parameters	Values
Laser power	3 kW
Welding speed	10 m/min
Wire feeding rate	4 m/min
Incident beam angle	22°
Wire feeding angle	20°
Protecting gas angle	20°
Focal position	Specimen surface
Protecting gas	Ar
Protecting gas flow rate	15 L/min

**Table 2:** Elastic parameters of the panel materials.

Panel Components	Young’s Modulus	Poisson’s Ratio
Skin	81 GPa	0.3
Stringer	81 GPa	0.3
Weld	81 GPa	0.3
Other components	72 GPa	0.3

**Table 3:** Failure loads for the experiment and FEM model.

	Failure Load (kN)	Percentage Difference (%) ^1^
Experiment	227.5	---
FEM	221.3	−2.7%

^1^ Percentage difference is the ratio of the difference between the failure load of the FEM model minus the failure load measured by the experiment and the failure load measured by the experiment.

## References

[1] Li Z., Gobbi S.L. (1997). Laser welding for lightweight structures. J. Mater. Process. Tech..

[2] Zink W. (1999). Advanced aircraft fuselage structures. The European Symposium on Assessment of Power Beam Welds.

[3] Rötzer I. (2005). Laser-beam welding makes aircraft lighter. Fraunhofer Mag..

[4] Kashaev N., Ventzke V., Gürel Çam (2018). Prospects of laser beam welding and friction stir welding processes for aluminum airframe structural applications. J. Manuf. Process..

[5] Zink W. (2001). Welding fuselage shells. Ind. Laser Solut. Manuf..

[6] Brenner B., Standfuβ J., Morgenthal L. (2004). New Technological Aspects of Laser Beam Welding of Aircraft Structures.

[7] Gupta R.K., Nayanb N., Nagasireesha G., Sharma S.C. (2006). Development and characterization of Al-Li alloys. Mater. Sci. Eng. A.

[8] Dursun T., Soutis C. (2014). Recent developments in advanced aircraft aluminum alloys. Mater. Des..

[9] Dittrich D., Standfuss J., Liebscher J., Brenner B., Beyer E. (2011). Laser beam welding of hard to weld Al alloys for a regional aircraft fuselage design—First results. Phys. Proced..

[10] Xia H.B., Tao W., Li L.Q., Tan C.W., Zhang K.P., Ma N.S. (2020). Effect of laser beam models on laser welding-brazing Al to steel. Opt. Laser Technol..

[11] Zhang X.Y., Yang W.X., Xiao R.S. (2015). Microstructure and mechanical properties of laser beam welded Al–Li alloy 2060 with Al–Mg filler wire. Mater. Des..

[12] Zhang X.Y., Huang T., Yang W.X., Xiao R.S., Liu Z., Li L. (2016). Microstructure and mechanical properties of laser beam-welded AA2060 Al-Li alloy. J. Mater. Process. Tech..

[13] Zhang Y.L., Tao W., Chen Y.B., Nan T.T. (2020). Effects of heat treatment on microstructure and mechanical properties of double-sided laser-welded AA2060/AA2099 T-joint. J. Mater. Process. Tech..

[14] Han B., Tao W., Chen Y.B., Li H. (2017). Double-sided laser beam welded T-joints for aluminum-lithium alloy aircraft fuselage panels: Effects of filler elements on microstructure and mechanical properties. Opt. Laser Technol..

[15] Ning J., Zhang L.J., Bai Q.L., Yin X.Q., Niu J., Zhang J.X. (2017). Comparison of the microstructure and mechanical performance of 2A97 Al-Li alloy joints between autogenous and non-autogenous laser welding. Mater. Des..

[16] Lynch C., Murphy A., Price M., Gibson A. (2004). The computational post buckling analysis of fuselage stiffened panels loaded in compression. Thin Walled Struct..

[17] Murphy A., Ekmekyapar T., Quinn D., Özakça M., Poston K., Moore G., Niblock J. (2014). The influence of assembly friction stir weld location on wing panel static strength. Thin Walled Struct..

[18] Wilson R., Murphy A., Price M.A., Glazebrook C. (2012). A preliminary structural design procedure for laser beam welded airframe stiffened panels. Thin Walled Struct..

[19] Han B., Chen Y.B., Tao W., Lei Z.L., Li H., Guo S., Li P. (2018). Nano-indentation investigation on the local softening of equiaxed zone in 2060-T8/2099-T83 aluminum-lithium alloys T-joints welded by double-sided laser beam welding. J. Alloys Compd..

[20] Murphy A., Price M., Lynch C., Gibson A. (2005). The computational post buckling analysis of fuselage stiffened panels loaded in shear. Thin Walled Struct..

[21] Tweedy B., Sellmeyer S., Jahn A., Burford D. Static strength comparison of riveted versus friction stir welded stiffened panels. Proceedings of the 47th AIAA/ASME/ASCE/AHS/ASC Structures, Structural Dynamics, and Materials Conference.

[22] Zhu S.H., Yan J.Y., Wang Y.Q., Tong M.B. (2015). Buckling and Postbuckling Experiments of Integrally Stiffened Panel Under Compression–Shear Loads. J. Aircr..

[23] Lei Z.K., Bai R.X., Tao W., Wei X., Leng R.J. (2016). Optical measurement on dynamic buckling behavior of stiffened composite panels under in-plane shear. Opt. Laser Eng..

[24] Hoffman E.K., Hafley R.A., Wagner J.A., Jegley D.C., Pecquet R.W., Blum C.M., Arbegast W.J. Compression Buckling Behavior of Large-Scale Friction Stir Welded and Riveted 2090-T83 Al-Li Alloy Skin-Stiffener Panels. http://www.researchgate.net/publication/24325063.

[25] Ge D.Y., Mo Y.M., He B.L., Wu Y.T., Du X.Z. (2016). Experimental and numerical investigation of stiffened composite curved panel under shear and in-plane bending. Compos. Struct..

[26] Liu X.Y., Han K., Bai R.X., Lei Z.K., Wang H. (2014). Buckling measurement and numerical analysis of M-type ribs stiffened composite panel. Thin Walled Struct..

[27] Wang Z.Y., Nguyen D.A., Barnes J.C. (2010). Some practical considerations in fringe projection profilometry. Opt. Laser. Eng..

[28] Zuo C., Huang L., Zhang M.L., Chen Q., Asundi A. (2016). Temporal phase unwrapping algorithms for fringe projection profilometry: A comparative review. Opt. Laser. Eng..

[29] Gorji N.E., Saxena P., Corfield M., Clare A., Rueff J.-P., Bogan J., González P.G.M., Snelgrove M., Hughes G., O’Connor R. (2020). A new method for assessing the utility of powder bed fusion (PBF) feedstock through life. Mater. Charact..

[30] ISO 13919-2 (2001). Welding-Electron and Laser Beam Welded Joints-Guidance on Quality Levels for Imperfections-Part 2, Aluminium and Its Weldable Alloys.

[31] Murphy A., Price M., Gibson A., Armstrong C.G. (2004). Efficient non-linear idealisations of aircraft fuselage panels in compression. Finite Elem. Anal. Des..

[32] Yang B., Shen L., Kang S.B., Elchalakani M., Nie S.D. (2018). Load bearing capacity of welded Q460GJ steel H-columns under eccentric compression. J. Constr. Steel Res..

[33] ASTM E 8M-04 (2004). Standard Test Methods for Tension Testing of Metallic Materials.

[34] ABAQUS (2014). ABAQUS/Standard User’s Manual and ABAQUS CAE Manual.

